# Cancer preventive effect of recombinant TRAIL by ablation of oncogenic inflammation in colitis-associated cancer rather than anticancer effect

**DOI:** 10.18632/oncotarget.23083

**Published:** 2017-12-07

**Authors:** Joo-Young Kim, Young-Mi Kim, Jong-Min Park, Young Min Han, Kang Choon Lee, Ki Baik Hahm, Suntaek Hong

**Affiliations:** ^1^ Laboratory of Cancer Cell Biology, Department of Biochemistry, School of Medicine, Gachon University, Incheon, Korea; ^2^ CHA Cancer Prevention Research Center, CHA Cancer Institute, CHA University, Seongnam, Korea; ^3^ College of Pharmacy, Sungkyunkwan University, Suwon, Korea

**Keywords:** TRAIL, colitis-associated colorectal cancer, cancer prevention, intestinal homeostasis, inflammasome

## Abstract

The potential of tumor necrosis factor-related apoptosis-inducing ligand (TRAIL) in inducing apoptosis is a hallmark in cancer therapeutics, after which its selective ability to achieve cell death pathways against cancer cells led to hope for recombinant TRAIL in cancer therapeutics. The present data from azoxymethane-initiated, dextran sulfate sodium-promoted colitis associated cancer (CAC) model strongly indicate the potential of rTRAIL in cancer prevention rather than in cancer therapeutics. Early treatment of rTRAIL significantly reduced colitis and CAC by inhibiting the recruitment of macrophages into the damaged mucosa and activating the scavenger activity with efferocytosis and the production of several growth factors. In contrast, late administration of rTRAIL as for anti-cancer effect did not decrease the initiation and development of CAC at all. Significant cancer preventing mechanisms of rTRAIL were identified. In the CAC model, anti-inflammation, regeneration, and efferocytosis was induced by treatment of TRAIL for 6 days, significant inhibitory activity was evident at 4 weeks and anti-oxidative and anti-inflammatory induction were noted at 12 weeks. Most importantly, TRAIL promoted tissue regeneration by enhancing the resolution of pathological inflammation through the activation of the NLRP3 inflammasome pathway. The results indicate that TRAIL reduces the induction of colitis and the initiation of CAC by inhibiting pro-inflammatory signaling and promoting tissue repair to maintain intestinal homeostasis through activation of the NLRP3 inflammasome. Therefore, TRAIL can be used as a chemopreventive agent against CAC, rather than as a therapeutic drug endowing apoptosis.

## INTRODUCTION

The induction of apoptosis in cancer cells has been the focus of many cancer therapeutic strategies involving apoptosis-inducing agents since avoiding apoptosis is one of the ley hallmarks of cancer. Many recombinant preparations of tumor necrosis factor (TNF)-related apoptosis-inducing ligand (TRAIL) or death receptor targeting monoclonal antibodies have been developed [1]. TRAIL is a member of the TNF ligand family that consists of 281 and 291 amino acid in the human and murine forms [2, 3]. TRAIL can bind to five different receptors: DR4 (TRAIL-R1), DR5 (TRAIL-R2), DcR1 (TRAIL-R3), DcR2 (TRAIL-R4) and osteoprotegerin. Binding sends different signals depending on the cognate receptor. Especially, DR4 and DR5 are pro-apoptotic receptors that rapidly induce apoptosis by activating caspase cascades [2–4]. There is an abundance of evidence implicating TRAIL in the acceleration of the apoptosis of neutrophils, leukocyte subsets, alveolar epithelial cells or other host cell-types in lipopolysaccharide (LPS)-induced lung injury and in cancer cells [5–8]. As exemplified by titles of publication like “Are we on the right TRAIL?” [9], “Targeting TRAIL towards the clinic” [10], “Exploring the TRAILs less travelled: TRAIL in cancer biology and therapy [11], further rational design of TRAIL-based therapy combined with other modality or advances in administration using nano- or cell–carriers is needed as cancer therapeutics [12].

In a preliminary study performed to measure the anti-cancer effect of rTRAIL in a gastric or colon cancer models, we recognized that the anti-cancer effect of rTRAIL involves multiple cancer pathways, including anti-inflammatory and anti-cancer actions of TRAIL on initiation, promotion, and progression of carcinogenesis as exemplified in *Helicobacter pylori*- or colitis-associated cancer (CAC). Especially inflammatory bowel disease (IBD), which includes ulcerative colitis and Crohn's disease, poses a significantly increased risk of developing colorectal dysplasia and cancer as compared to the general population [13–15]. Although the exact molecular mechanisms related with CAC are not fully understood, it is generally believed that appropriate levels of anti-inflammation are protective, while excessive inflammation is pathological for IBD and CAC [16–18]. When the homeostasis in intestinal mucosa is disrupted, the commensal bacterial are the basis of IBD pathogenesis and promote CAC [18].

In this study, for the first time, we found that rTRAIL preempted apoptosis, subsequently mitigated inflammation, afforded optimal efferocytosis, spurted regeneration, and achieved intestinal homeostasis in addition to apoptosis to inflammatory cells, and concluded that rTRAIL significantly mitigated longstanding mutagenic inflammation-based cancer rather than having anti-cancer effects as evidenced earlier application of rTRAIL prevented CAC, while late administration did not.

## RESULTS

### Early administration of rTRAIL prevented colitis-associated carcinogenesis

**Figure 1:**
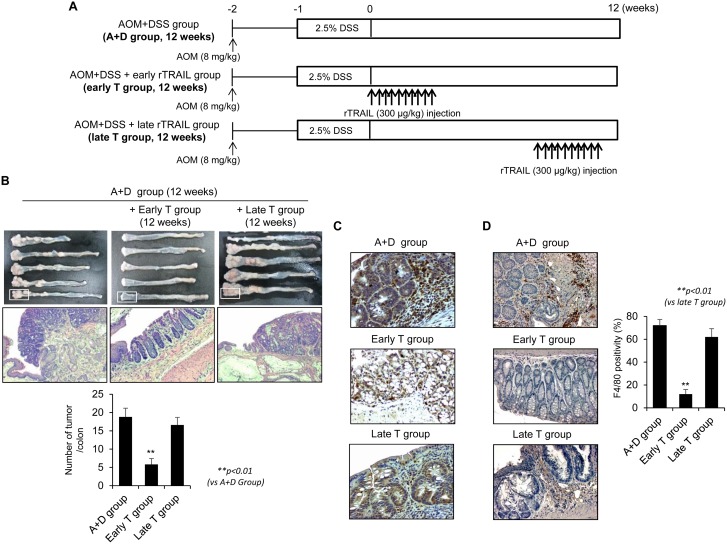
TRAIL has a protective effect in AOM/DSS-induced CAC only by early administration (**A**) C57BL/6 mice were treated with 2.5% DSS and 8 mg/kg AOM (*N* = 8 mice per group, A+D Group) and injected with TRAIL 10 times within 10 days at early (0 week, early T Group) and late (10 week, late T Group) with 300 μg/kg dose. (**B**) After 12 weeks, colons were obtained and observed the severity of CAC. After washing the colon tissues, number of tumors was counted and compared with TRAIL-treated groups. (**C**) Neoplastic colon tissues were immunostained with β-catenin antibody (upper lane). (**D**) Tumoric parts of A+D group or T group were immunostained using F4/80 antibody to check the infiltration of macrophages. The positive cells were counted and compared with TRAIL-treated groups. Results are presented as mean ± SD from three independent experiments.

Since rTRAIL has been known as anti-cancer drug that selectively kills several kinds of cancer cells without damaging normal cells [6], we first compared the influence of rTRAIL in progression of azoxymethane (AOM)-initiatedand dextran sulfate sodium (DSS)-promoted colon carcinogenesis. To test the therapeutic activity of rTRAIL, we treated the rTRAIL at different times coinciding with DSS administration (early T group) or administration 10 weeks after AOM/DSS (late T group). Mice were administrated AOM/DSS and further treated with 10 times of rTRAIL (300 μg/kg) at beginning 0 and 10 weeks later (Figure 1A). The AOM-initiated, DSS-promoted model led to the significant development of colorectal tumor after 12 weeks in all seven mice of the control group (Figure 1B). Unexpectedly, only 25% (2 of 8) mice developed colorectal tumors in the group treated with rTRAIL coinciding with DSS administration (early T group), and the tumor incidence did not changed at all in mice treated with rTRAIL beginning 10 weeks after DSS administration (late T group). Gross and microscopic analyses revealed that mice injected with rTRAIL beginning 10 weeks after AOM/DSS administration developed many tumors, similar to the untreated group, while tumor development was totally suppressed in the early treated group (*p <* 0.01; Figure 1B). Examination of further time points indicated the efficacy of rTRAIL, administered at 5 weeks after DSS, was marginal between 0 and 10 weeks treatment and better than control group in tumorigenesis (Supplementary Figure 1A and 1B). The β-catenin immunostaining revealed significantly different labeling of aberrant β-catenin cryptic gland, and expressions and location of β-catenin between the early and late groups (Figure 1C). Emergence of β-catenin accumulated aberrant cryptic glands (BCAC) were reported to be the prime events noted in CAC model, but earlier administration of rTRAIL significantly prohibited the emergence of BCAC, signifying rTRAIL efficiently inhibited the activation of β-catenin signaling. To examine whether TRAIL inhibits the β-catenin signaling directly or indirectly, we checked the dissociation and translocation of β-catenin in the presence of TRAIL (Supplementary Figure 1C and 1D). Treatment of TRAIL induced the interaction of β-catenin with Axin and GSK3β and thereby inhibited the translocation of β-catenin into nucleus. This result suggests that TRAIL may directly block the β-catenin signaling by activating GSK3β-mediated β-catenin degradation. Macrophage analysis using F4/80 immunostaining also revealed a significant difference between the groups (Figure 1D), indicating preventive activity, rather than therapeutic activity, of rTRAIL for CAC.

### rTRAIL inhibited either initiation or progression of CAC by suppressing β-catenin signaling

**Figure 2:**
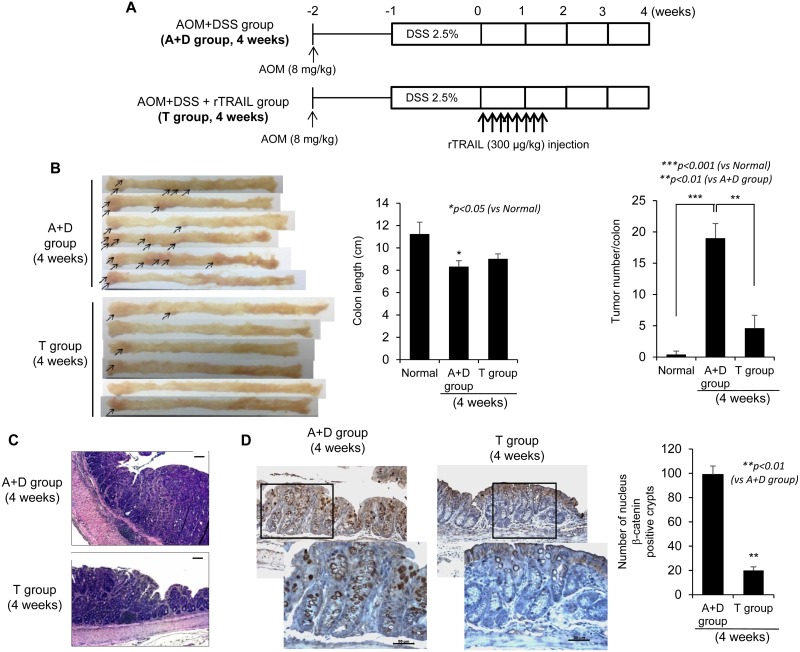
TRAIL inhibits initiation of CAC by blocking activation of β-catenin (**A**) C57BL/6 mice are treated with 2.5% DSS and 8 mg/kg AOM (*N* = 6 mice per group) and injected TRAIL (300 μg/kg) for 10 times within 10 days. The mice were sacrificed after 4 weeks to prepare the colon tissues. (**B**) After 4 weeks, colons were obtained and measured length and number of tumors. The arrows indicated the formed tumors. (**C**) Neoplastic colon tissues were stained with H&E. Scale bar denotes 100 μm. (**D**) To confirm the tumorigenesis of dysplastic residue, colon tissues of each group were immunostained with β-catenin. Scale bar denotes 50 μm. The positive cells were counted and compared with control groups. Results are presented as mean ± SD from three independent experiments.

Given the significant achievements in cancer preventive effect of rTRAIL in early intervention, we repeated the experiment examining the effect of rTRAIL on tumor-associated signaling 4 weeks after AOM/DSS administration (Figure 2A). Mice were treated with TRAIL soon after administration of AOM/DSS and were sacrificed after 4 weeks to check the activation of oncoproteins. As expected, DSS in combination with AOM led to significant development of small colorectal tumors (proven to be colon dysplasia on histology, Figure 1C) after 4 weeks. Even at 4 weeks, rTRAIL-treated mice showed significantly decreased development of colorectal tumors compared with control mice (Figure 2B). Since colorectal tumor development was associated with DSS administration (Figure 2B), colon length was significantly decreased in the control group, but not in the rTRAIL treated group (*p <* 0.05). There was significant decrease in the number of tumors with rTRAIL treatment (*p <* 0.01, Figure 2B). The tumors of control group were larger than that of TRAIL-treated group as revealed with H&E staining (Figure 2C). To gauge representative genetic changes in tumorigenesis, we performed β-catenin immunotaining. Four weeks of AOM/DSS administration, β-catenin expression was significantly increased (Figure 2D). Nuclear translocation, defined as aberrant β-catenin cryptic glands, was evident in the control group but was significantly decreased in rTRAIL treated group (*p* < 0.01, Figure 2D). These data demonstrate that earlier rTRAIL administration inhibits the development of CAC tumorigenesis by blocking the activation and translocation of β-catenin-associated proliferative and mutagenic signals as well as mutagenic inflammatory activities.

### rTRAIL mitigated AOM/DSS-induced colitis by either interrupting inflammatory signaling or spurting regeneration

**Figure 3:**
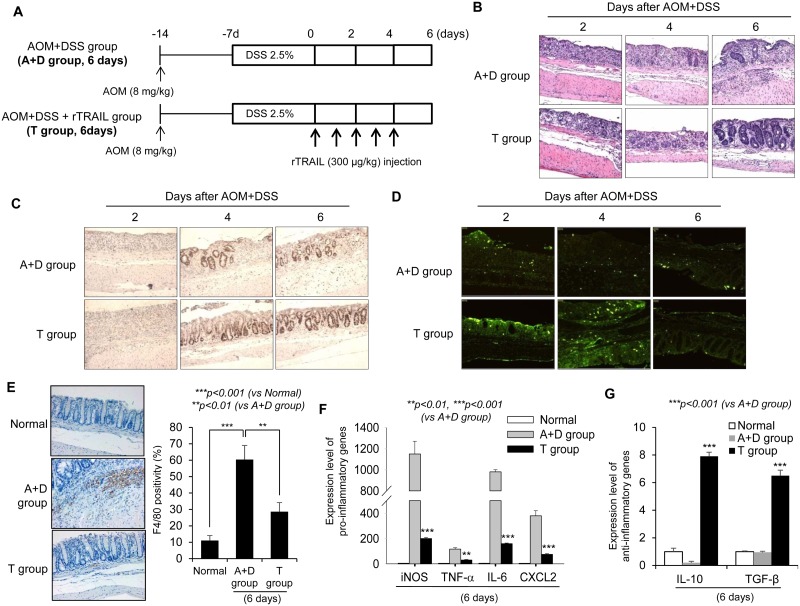
TRAIL reduces AOM/DSS-induced inflammatory signaling (**A**) C57BL/6 mice were treated with 2.5% DSS and 8 mg/kg AOM (*N* = 8 mice per group) and administrated TRAIL (300 μg/kg) every day. Colons were obtained at day 2, 4 and 6. (**B**) Damaged colon tissues are stained with H&E of PBS-treated control (top) or TRAIL-treated mice (bottom) at each time points. (**C**) Similar residue of inflamed colon tissues are immunostained with PCNA. The numbers of positive cells as proliferating cells are significantly increased in TRAIL-treated group. (**D**) The apoptotic cells were detected with TUNEL assay. (**E**) The number of recruited macrophages in damaged tissue was measured with staining of F4/80 and compared with control group. (**F** and **G**) The expression level of pro- or anti-inflammatory cytokines was measured with qRT-PCR. The relative level of each gene was normalized with housekeeping *Cyclophilin* gene. Results are presented as mean ± SD from three independent experiments.

Given that the earlier injection of rTRAIL suppressed formation of AOM/DSS-induced colorectal tumors, while no therapeutic/preventive effects were evident with later administration, we hypothesized that TRAIL has preventative activity early following initiation of colitis. To determine the effect of rTRAIL on early inflammation, we examined the AOM/DSS-induced colitis mouse model and harvested the colon tissues after day 2, 4, and 6 (Figure 3A). Significant loss of crypts accompanied with severe colon ulceration was noted in both control and rTRAIL treatment group on day 2. However, at days 4 and 6, there were higher activities of mucosal regeneration in TRAIL-treated group mice compared with control group (Figure 3B). Compared to AOM/DSS control group, the mean colon length was significantly decreased (Supplementary Figure 2A), and the degree of ulceration and colon inflammation was markedly decreased in rTRAIL-treated group (Figure 3B and 3C). The structure of mucosal tissues was collapsed by DSS exposure since day 2, and TRAIL-treated mucosa showed increased epithelial cell proliferation as accessed by proliferating cell nuclear antigen (PCNA) immunohistochemistry staining compared to control mice (Figure 3C). To exclude the possibility of TRAIL-mediated proliferation of colon epithelial cell, we checked the cell proliferation using MTT assay in the presence of TRAIL (Supplementary Figure 2B). Treatment of TRAIL did not enhance the proliferation of colon epithelial cells. This result suggest that TRAIL may promote the proliferation of colon epithelial cells through another way, such as removal of damaged cell or change of microenvironment. In the DSS-induced inflamed situation, colonic homeostasis is disrupted by external pathogens from damaged epithelium, and recruited immune cells die and are taken up by macrophages. A recent report reported that TRAIL may block the inflammation by due accelerating neutrophil apoptosis [19]. We measured the apoptotic cells in damaged mucosa using the TUNEL assay. The TRAIL-treated group showed numerous apoptotic cells compared with the control group (Figure 3D). Consistent with the TUNEL assay, TRAIL treatment markedly induced the activation of caspase cascades (Supplementary Figure 2C). As inflammation developed, DSS treatment increased the recruitment of macrophages into damaged intestinal tissues (Figure 3E). However, the recruited macrophages were significantly decreased in TRAIL-treated colon tissues (*p <* 0.01). Also, pro-inflammatory cytokines including iNOS, TNF-α, IL-6 and CXCL2 were downregulated in TRAIL-treated mice (*p <* 0.001, Figure 3F). In contrast, the anti-inflammatory cytokines IL-10 and TGF-β were significantly increased in TRAIL-treated intestinal tissues (*p <* 0.001, Figure 3G). The collective data strongly suggest that rTRAIL erased the damaged intestinal epithelial cells via reducing inflammation and suppressed the recruitment and activation of macrophages in the mucosa accompanied with induction of anti-inflammatory cytokines. To identify the source of anti-inflammatory cytokines in TRAIL-treated group, we checked the detail subtypes of infiltrated cells using immunohistochemistry. Interestingly, M2 macrophages (CD163) were more increased in TRAIL-treated group compared to AOM/DSS group (Supplementary Figure 2D). However, there is no detectable level of regulatory T cells (FoxP3) in colon tissues. These results suggest that increase of anti-inflammatory cytokines in TRAIL-treated group may originate from the promotion of recruitment or differentiation of M2 macrophages by TRAIL.

### TRAIL inhibited inflammatory signaling by inducting preemptive apoptosis and activation of efferocytosis in macrophages

**Figure 4:**
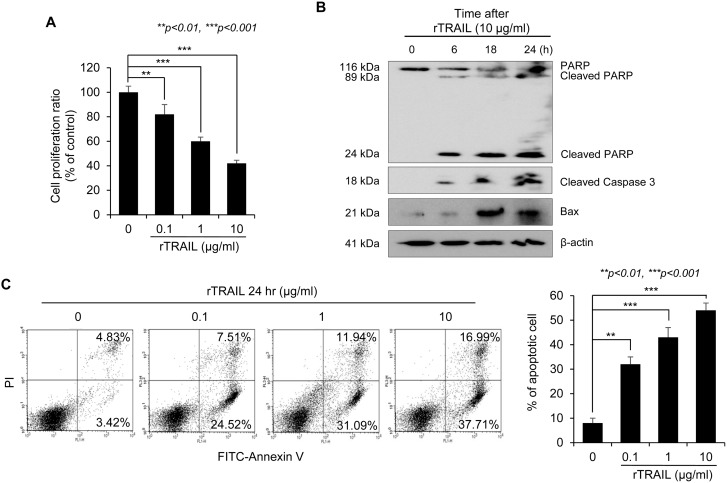

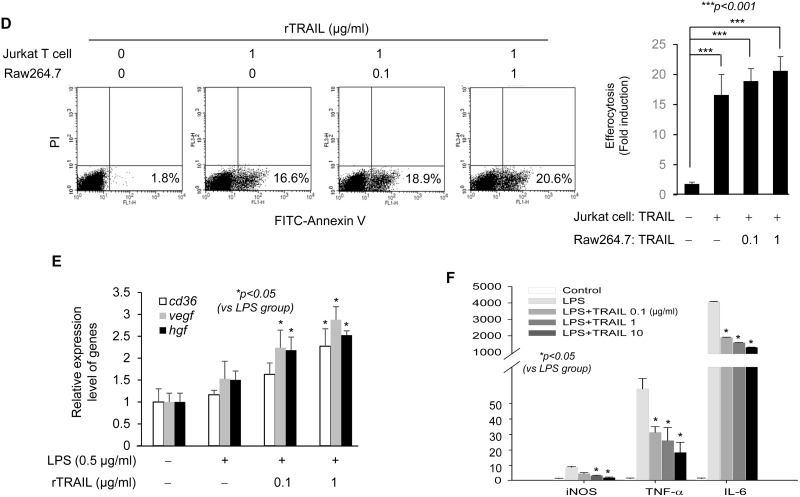
TRAIL induces apoptosis of immune cells and efferocytic activity of macrophages (**A**) The cell proliferation rate of Jurkat T cells was measured with MTT assay after treatment of TRAIL. After 24 hr, the proliferating cells were compared with non-treated group. (**B**) Cell lysate were prepared from Jurkat T cells that were treated with TRAIL (10 μg/ml) for 6, 18 and 24 hr and the expression level of apoptosis related proteins such as PARP, Caspase 3 and Bax were detected by specific antibodies. The loading amount was normalized with β-actin. (**C**) To confirm the TRAIL-mediated apoptosis, Jurkat T cells were treated with various concentrations of TRAIL (0. 0.1, 1, 10 μg/ml) for 24 hr. Then, TRAIL-induced apoptotic Jurkat T cells were detected by FITC-conjugated Annexin V kit. The percentage of dead cells was counted and was compared with live cells. (**D**) To test the activity of efferocytosis in macrophage, FITC-Annexin V-stained Jurkat T cells were treated with TRAIL and were co-cultured with Raw246.7 macrophage cells pretreated with TRAIL. The activity of phagocytosis was measured by detection of fluorescence using flow cytometry. (**E**) The mRNA expression of scavenger receptor (CD36) and growth factors (VEGF and HGF) was checked with qRT-PCR after treatment of LPS and TRAIL. The relative level of each gene was normalized with housekeeping *Cyclophilin* gene. Results are presented as mean ± SD from three independent experiments. (**F**) To examine the effect of TRAIL on LPS-induced expression of inflammatory genes, Raw246.7 cells were treated with different concentration of TRAIL (0, 0.1, 1, 10 μg/ml) in the presence of LPS (0.5 μg/ml) for 3 hr. Total RNA was isolated and qRT-PCR for various inflammatory mediator genes, such as iNOS, TNF-α, and IL-6 mRNA was performed. The relative level of each gene was normalized with housekeeping *Cyclophilin* gene. Results are presented as mean ± SD from three independent experiments.

To explain the mechanism how rTRAIL lessened mutagenic inflammation, we first checked the apoptotic cell death of inflammatory cells. TRAIL suppressed the growth of Jurkat T cells in a dose-dependent manner (Figure 4A). TRAIL-mediated apoptosis of Jurkat T cells was also detected with Western blot by checking the temporal change of apoptosis signal proteins including PARP, caspase-3 and Bax (Figure 4B). Flow cytometry using PI and Annexin V staining also revealed the significant induction of apoptosis of T cells by rTRAIL (Figure 4C). Next, we used apoptotic Jurkat T cells to explain how TRAIL induces the phagocytic activity of macrophages with the efferocytosis engulfment assay. When RAW264.7 macrophages were incubated with apoptotic Jurkat T cells, the macrophages could engulf the dead Jurkat T cells with TRAIL stimulation. Engulfment of the TRAIL-stimulated macrophages was enhanced with TRAIL treatment in a dose dependent manner (Figure 4D). Moreover, the expression of CD36, which is a scavenger receptor, was induced by TRAIL in LPS-stimulated Raw264.7 cells and other growth factors (vascular endothelial growth factor and hepatocyte growth factor) that promote proliferation of epithelial cells displayed a similar tendency (Figure 4E). Finally, qRT-PCR was performed to check the influence of rTRAIL on the mRNA expression of inflammatory mediators, such as iNOS, TNF-α and IL-6. rTRAIL led to significant mitigation of LPS-induced iNOS, TNF-α and IL-6 in colon epithelial cells (Figure 4F). These results indicated that TRAIL induced phagocytic activity of macrophages in order to scavenge dead immune cells, preemptive apoptosis and associated epithelial regeneration for tissue repair.

### TRAIL led to prevention of CAC through activating NLRP3 inflammasome signaling

**Figure 5:**
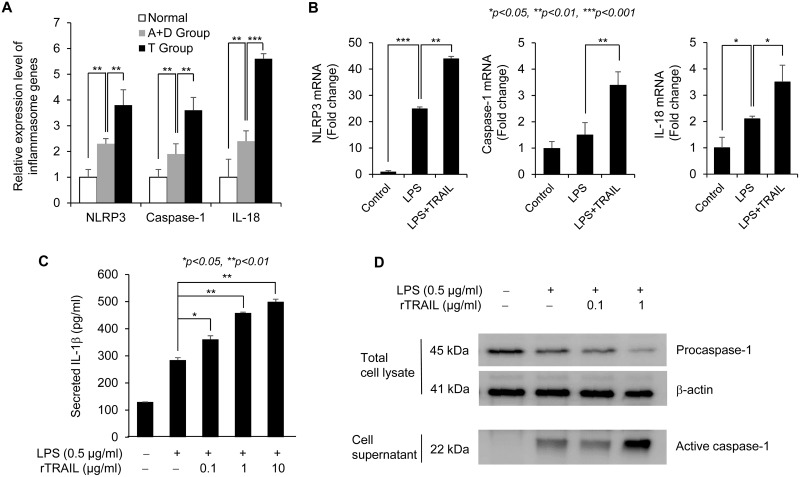
TRAIL upregulates the NLRP3 inflammasome signaling (**A**) To confirm the microarray data, we checked the mRNA expression level of representative signal transducer of NLRP3 inflammasome pathway, such as *NLRP3*, *Caspase-1* and *IL-18*, using qRT-PCR with mouse tissues. The relative level of each gene was normalized with housekeeping *Cyclophilin* gene. Results are presented as mean ± SD from three independent experiments. (**B**) To validate the upregulation of inflammasome-related genes by TRAIL, we checked the mRNA expression level of representative signal transducer of NLRP3 inflammasome pathway such as *NLRP3*, *Caspase-1*, and *IL-18* using qRT-PCR with treatment of LPS with/without TRAIL in Raw246.7 cells. The relative level of each gene was normalized with housekeeping *Cyclophilin* gene. Results are presented as mean ± SD from three independent experiments. (**C**) To validate the promotive effect of TRAIL on LPS-induced activation of inflammasome signaling, Raw246.7 cells were treated with different concentrations of TRAIL (0, 0.1, 1, 10 μg/ml) in the presence of LPS (0.5 μg/ml) for 8 hr. Secretion of IL-1β was induced by addition of ATP (5 mM) for 1 hr. Amount of secreted IL-1β was measured with ELISA after harvesting culture supernatant. (**D**) To measure the inflammasome activity, Raw246.7 cells were seeded into 6 well plate and treated with LPS alone (0.5 μg/ml) or cotreated with different amounts of TRAIL (0, 0.1, 1 μg/ml) for 4 hr and followed by addition of ATP (5 mM) for 1 hr. Then, culture supernatants were precipitated by adding methanol and chloroform and following centrifugation. The protein pellets were resuspended in loading buffer and analyzed by SDS-PAGE with caspase-1 antibody. Total cell lysates were used for detection of procaspase-1 and β-actin as normalization control.

To determine which signaling pathway is related with the protective effect of TRAIL on CAC, we analyzed the differential gene expression in acute colitis samples treated with AOM/DSS for 6 days with/without TRAIL using the Agilent mouse 8 × 60 k microarray and identified the related signal pathways using the Ingenuity IPA program. As shown in Supplementary Figure 3, several apoptosis pathways were activated in the TRAIL-treated group. Since recent studies suggest that physiologic levels of inflammation are protective and that excessive inflammation is deleterious, we focused on the intestinal homeostasis pathway that is regulated by the inflammasome caspase involved in the resolution of inflammation [20, 21]. The NLRP3 inflammasome pathway was strongly related with treatment of TRAIL in acute colitis model (Figure 5A). We confirmed that the major signal mediators NLRP3, caspase-1 and IL-18 were upregulated in the TRAIL-treated group compared to normal or AOM/DSS-treated group. TRAIL-dependent upregulation of inflammasome mediators was also confirmed in LPS-treated macrophage cells (Figure 5B). To validate the TRAIL-mediated activation of inflammasome signaling, secreted IL-1β was measured in Raw264.7 cells with ELISA (Figure 5C). Consistent with mRNA expression of inflammasome mediators, secretion of IL-1β was increased by costimulation with LPS and TRAIL. To measure the inflammasome activity in another way, activated caspase-1 was compared in LPS and TRAIL-treated groups. As shown Figure 5D, treatment of TRAIL enhanced the LPS-induced secretion of active caspase-1. Taken together, these results support the view that TRAIL increases epithelial repair after injury by upregulating NLRP3 inflammasome signaling in chronic journey of CAC development.

## DISCUSSION

This study is the first description of strong cancer prevention effect of rTRAIL associated with a potent initial abrogation of mutagenic inflammation and proliferation control, while rTRAIL administration later in tumor formation being ineffective, lower than expectation as anti-cancer effect of rTRAIL. When compared with the outcome of the established AOM initiated-, DSS promoted CAC model, early intervention using TRAIL significantly prevented CAC, with <25% incidence with mild dysplasia. In contrast, late administration of rTRAIL did not show any cancer prevention or anti-cancer effects (Figure 1). Subsequent observations at 4 weeks and 6 days after AOM/DSS-induced colitis and colon tumorigenesis demonstrated that earlier administration of rTRAIL afforded significant anti-tumorigenic actions. We conclude rTRAIL administration can be applied for chemopreventive purposes rather than for its current use as an anti-cancer agent. While TRAIL-induced programed necrosis might be effective in eliminating tumor cells [22], TRAIL-secreting stem cells promote cancer cell death [23, 24], and TRAIL or TRAIL-T cells sensitize cancer cell death [25, 26], our study is, to our knowledge, the first report of a cancer prevention role of TRAIL.

Sequential accumulation of somatic mutations and clonal expansions occur in IBD-associated CAC patients [27] and the chronic inflammatory microenvironment spurs the accelerated epithelial cell turnover by maintaining high concentration of inflammatory cytokines, such as IL-6, TNF-α and CXCL2, and the continued exposure to oxidative stress promotes neoplastic transformation of colon tissues [28–30]. Thus, the top-down based earlier application of rTRAIL should be considered for chemoprevention. Especially, in inflammation-based carcinogenesis, the innate immune system is the most important defense mechanism against invading microbial organisms in intestinal mucosa homeostasis. Especially, pattern recognition receptors recognize bacterial intrusion caused by the damaged epithelial barrier and is critical in tissue repair, which results in a robust innate immunity network at intestinal mucosal surfaces [31, 32]. Notably, in several animal models involving deleted Toll-like receptor (TLR)-IL-1β, IL-18 and MyD88, a markedly enhanced susceptibility in damaged epithelium with either irradiation or administration of DSS has been described [33, 34]. In addition to TLR, the inflammasome is a key component of the cytosolic surveillance complex that controls the maturation of pro-inflammatory cytokines including IL-1β or IL-18 [35]. Single-nucleotide polymorphisms of human NLRP3 gene have been related with susceptibility to Crohn's disease and the IL-18 cytokine has been shown to contribute to intestinal epithelial cell regeneration to chronic inflammation in IBD [18].

**Figure 6:**
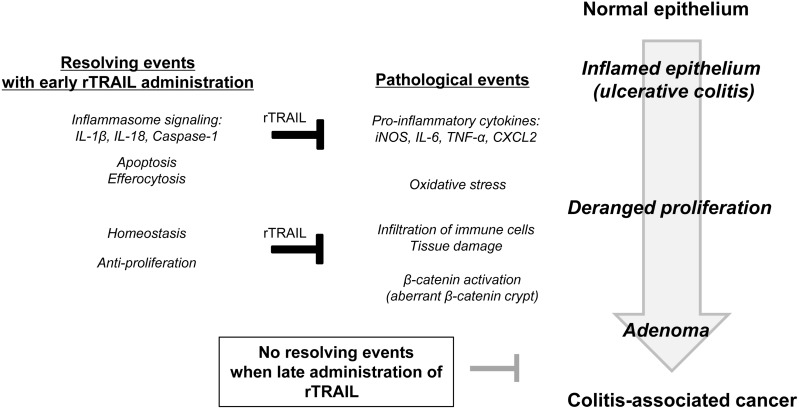
Schematic diagram showing preventive activity of TRAIL in CAC In the process of colitis-associated inflammation, pro-inflammatory cytokines are upregulated to induce the tissue damage and lead to abnormal activation of β-catenin signaling. To resolve these pathological processes, TRAIL induces the gene expressions involving in inflammasome signaling, enhances the apoptosis of immune cells and promotes the efferocytosis of macrophages. Based on these data, TRAIL can be used as preventive agent of CAC by ablation of oncogenic inflammation rather than therapeutic drug.

TRAIL is highly expressed in immune cells and is involved in fighting infection and modulating immune response. *Listeria* infection in wild type mice is reportedly much more dangerous than that observed in TRAIL-deficient mice, with cell death reduced in lymphoid and myeloid cells [36]. In addition, neutrophil-derived TRAIL can induce the death of DR5-positive macrophages and can enhance the rapid clearance of bacteria in pneumococcal pneumonia [37]. In this study, TRAIL displayed a protective function against DSS-promoted colitis by several mechanisms (Figure 6). First, TRAIL inhibits the recruitment of activated macrophages, leading to low-level inflammation and the acceleration of efferocytic activity of dead immune cells. Second, TRAIL promotes the regeneration of intestinal epithelium through increased growth factors. Colons inflamed by AOM/DSS increase the infiltration of inflammatory cells, such as CD3^+^ T lymphocytes and F4/80^+^ macrophages; TRAIL reduced the recruitment of these immune cells by blocking the expression of inflammatory cytokines. TRAIL also enhances the resolution of inflammation through upregulation of inflammasome-related genes. Consistent with our results, TRAIL-R knockout mice showed a high incidence of DSS-induced colitis and CAC compared to wild-type littermates through higher expression of inflammatory cytokines and marked infiltration of immune cells [25]. These data indicate that TRAIL-R signaling functions as a gatekeeper in chemical-induced colon damage and mucosal immune reactions. In another study, loss of TRAIL-R significantly reduced the lymphoma-free survival and showed higher metastatic potential and defective apoptosis [38]. Increased numbers of hepatic cancer were also observed with diethylnitrosamine-induced model in TRAIL-R deficient mice. Based on these data, we propose that TRAIL efficiently prevents CAC progression through the TRAIL-R-mediated signaling pathway by modulating mucosal immune responses.

Because TRAIL has tumoricidal activity on various cancer types, many clinical trials have tested the efficacy as therapeutic agent with recombinant human TRAIL (Dulanermin). TRAIL alone or combination with conventional chemotherapeutics or biologics has been evaluated for treatment of advanced cancer including lymphoma, colorectal cancer and non-small cell lung cancer [39–41]. Although treatment of rTRAIL was well tolerated by cancer patients, clinical trials failed due to low sensitivity of TRAIL-based therapies. To overcome short half-life or nonspecific interaction of TRAIL, modified TRAIL variants were developed to improve its cytotoxic activity [42–44]. Despite good results *in vitro*, there are still barriers to clinical use. These discrepancies may originate from the limitation of tumoricidal activity of TRAIL. Previous clinical trials mainly focused on removal of established cancers, not prevention of carcinogenesis. We also found that late treatment of TRAIL did not show any therapeutic activity compared to early treatment (Figure 1). This result suggests that TRAIL can be used as a chemopreventive agent, rather than as a therapeutic drug. By preventing the pathological inflammatory signaling and tissue damage, TRAIL could prevent the development and progression of colorectal cancer. Although it is difficult to use TRAIL as a chemopreventive agent for human cancers, it will be possible to screen for drugs that activate TRAIL receptor signaling to induce the apoptosis and remove the damaged cells [45].

Here, we describe the novel regulatory functions of TRAIL in development of CAC through either efficient suppression of mutagenic inflammatory signaling or activation of scavenger pathway and regenerative action. Most importantly, TRAIL prevents the initiation and progression of CAC by early treatment, but not by late treatment by activating inflammasome pathway, which mediates host defense against microbial pathogens or tissue homeostasis [46]. This data indicate that TRAIL has strong chemopreventive activity against CAC by removing the inflammation-induced damaged cells to suppress the transformation of intestinal epithelial cells and promoting the tissue repair with upregulation of resolution-related genes. Therefore, it should prove to be a valuable approach to screen the chemicals or natural products which activate or enhance the TRAIL-mediated apoptotic signaling to prevent the development of CAC.

## MATERIALS AND METHODS

### Animal model of colitis-associated colorectal carcinogenesis

To induce colitis-associated colon tumors, 8-wk-old C57BL/6 mice (Orient, Seoul, Korea) were treated with AOM (Sigma-Aldrich, St. Louis, MO) followed by administration of DSS. Briefly, a single dose (8 mg/kg) of the mutagenic agent, AOM, was injected intraperitoneally followed by one cycle (5 days) of 2.5% DSS (molecular mass, 40 kDa; MP Biomedicals, Santa Ana, CA) dissolved in sterile distilled drinking water. After treatment, mice were daily injected with soluble TRAIL (300 μg/kg, [42]) for 10 times at early (0 weeks), middle (5 weeks) and late (10 weeks) time points. After 5 or 12 weeks, mice were sacrificed and colon tissue was collected, opened longitudinally, and processed for histology or gene expression analysis. All animal experiments were performed with protocols approved by the Center for Use and Care of Animals of Gachon University (Incheon, Korea).

### Cell culture

RAW264.7 macrophage cells and Jurkat T cells were obtained from the American Type Culture Collection (ATCC, Manassas, VA) and were cultured as mentioned. Cells were grown in DMEM supplemented with 10% fetal bovine serum, streptomycin and penicillin in a CO_2_ incubator. To analyze gene expression, the cells were seeded in wells of 6-well plates and stimulated with LPS (0.5 μg/ml) and TRAIL (0.1, 1 and 10 μg/ml) for 6 or 24 hr.

### Western blotting

Protein concentration was calculated with the BCA protein assay (Pierce, Rockford, IL). SDS-PAGE was used to separate the tissue or cell proteins and the re3solved proteins were transferred to polyvinylidene fluoride membranes. After washing, membranes were blocked with 5% skim milk for 30 min and then incubated for 3 hr with the primary antibody to detect Caspase 3, Bax (Cell Signaling Technology, Beverly, MA), Caspase 8 and PARP (Santa Cruz Biotechnology, Santa Cruz, CA). Proteins were visualized by incubation with a secondary antibody conjugated with horseradish peroxidase and chemiluminescence, according to manufacturer's protocols (Pierce).

### Histological analysis and immunohistochemistry

Paraffin-embedded mouse colon tissues were mounted on slides coated with silane (Dako, Santa Clara, CA) and stained with hematoxylin for histological analysis. Hematoxylin-eosin staining was performed according to standard protocols. For immunohistochemistry, slides were deparaffinized in xylene and rehydrated in a series of graded alcohols, and the antigen was retrieved in 0.01 mM sodium citrate buffer. Slides were treated with 3% of hydrogen peroxide to block endogenous peroxidase. After incubation with specific antibody, the Dako REAL EnVision system was used as detection system according to the manufacturer's protocols (Agilent, Santa Clara, CA). The reaction was evaluated by use of the appropriate substrate/chromogen reagent and Mayer's hematoxylin (Sigma-Aldrich) was used for counterstaining.

### Measurement of gene expression with PCR system

Total RNA was isloated from cells using Trizol-reagent (Invitrogen, Carlsbad, CA) and cDNA generated using PrimeScript 1st strand cDNA Synthesis Kit (6110A, TaKaRa, Shiga, Japan). Samples were analyzed with quantitative real time PCR (qRT-PCR) using the C1000-Thermal Cycler (Bio-Rad Laboratories, Irvine, CA) with SYBR^®^ Premix Ex Taq II (TaKaRa) and normalized with *cyclophilin* as housekeeping gene. Gene specific primers for PCR are summarized in Supplementary Table 1.

### Detection of cell apoptosis and efferocytosis

To check the effect of TRAIL on immune cells, Jurkat T cells were treated with different concentrations of TRAIL for 24 hr. Apoptosis was measured with annexin V and propidium iodide (PI) staining using flow cytometry (FACSCalibur, BD Biosciences, San Jose, CA). The apoptotic activity of TRAIL was compared with non-treated control cells. To determine the *in vitro* efferocytic activity of macrophages, RAW264.7 cells were co-incubated for 1 hr with Jurkat T cells (stained with FITC-conjugated annexin V) undergoing apoptosis. To remove the non-engulfed apoptotic Jurkat T cells, RAW264.7 cells were washed three times with PBS and the proportion of RAW264.7 cells containing apoptotic Jurkat T cells (FITC-positive cells) was assessed by flow cytometry and fluorescent microscopy. Apoptosis of Jurkat T cells was induced by TRAIL (10 μg/ml), followed by incubation for 8 hr at 37°C in an atmosphere of 5% CO_2_.

### Microarray analysis

Total RNA (1 μg) was labeled and amplified using the ULS aRNA labeling kit (Kreatech Diagnostics, Amsterdam, Netherlands). The Cy5 or Cy3-labeled aRNAs were hybridized with mouse whole genome 8 × 60 k arrays (Agilent Technologies) and gene expression levels were calculated with Feature Extraction v10.7.3.1 (Agilent Technologies). The data were processed using the median Polish normalization method and GeneSpring GX 11.0 software (Agilent Technologies). The normalized and log-transformed intensity values were then analyzed using GeneSpring GX 11.0. Ingenuity Pathway Analysis (IPA, Qiagen) was used for identification of significant pathway related with differentially expressed genes.

### Statistical analysis

Results from at least three separate experiments are expressed as mean ± S.D. The Student's *t*-test was used for comparisons between control and treatment groups. Differences between samples were considered significant for *P*-values < 0.05.

## SUPPLEMENTARY MATERIALS FIGURES AND TABLES



## Abbreviations

TRAIL: Tumor necrosis factor-related apoptosis-inducing ligand
rTRAIL: Recombinant TRAIL
CAC: colitis-associated cancer
IBD: Inflammatory bowel disease
AOM: Azoxymethane
DSS: Dextran sulfate sodium
TLR: Toll-like receptor
SOCS: Suppressors of cytokine signaling
