# Supplementary material for: Cancer preventive effect of recombinant TRAIL by ablation of oncogenic inflammation in colitis-associated cancer rather than anticancer effect
Source: Oncotarget. 2017 Dec 7;9(2):1705–16. doi: 10.18632/oncotarget.23083 (PMC5788592; doi:10.18632/oncotarget.23083)
Supplement: Supplementary file 1 [file oncotarget-09-1705-s001.pdf]

Cancer preventive effect of recombinant TRAIL by ablation of oncogenic inflammation in colitis-associated cancer rather than anticancer effect

SUPPLEMENTARY MATERIALS

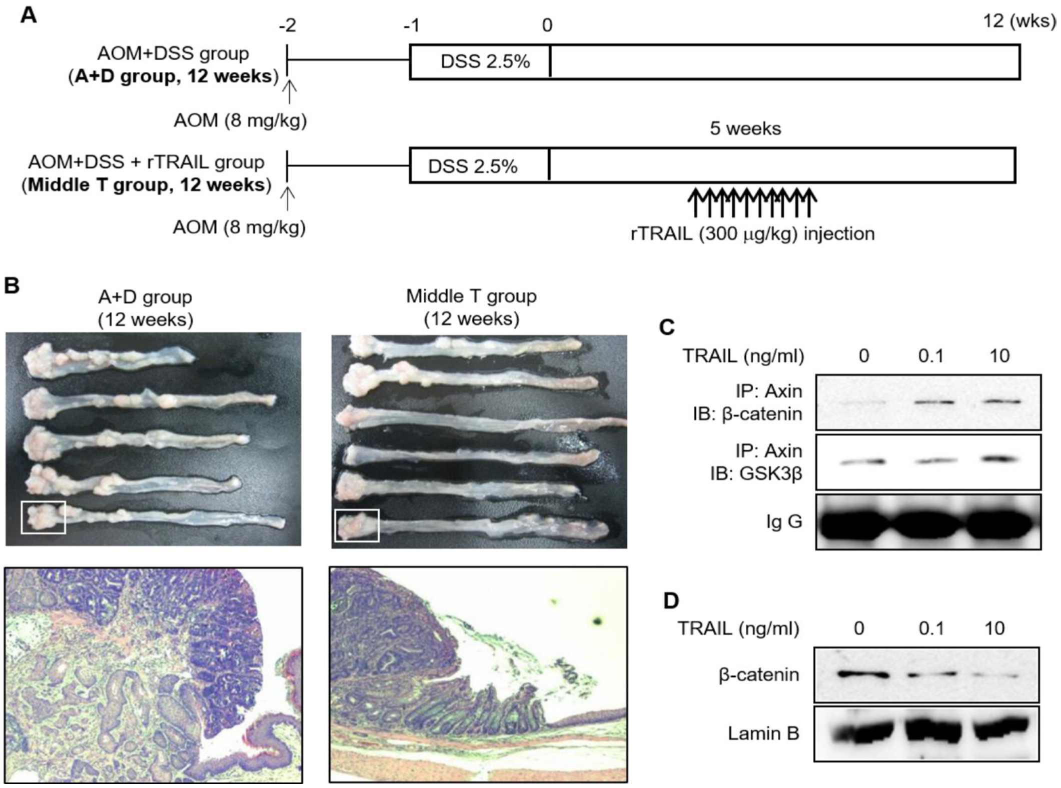

Supplementary Figure 1: TRAIL has an intermediate effect in AOM/DSS-induced CRC by middle administration. (**A**) C57BL/6 mice were treated with 2.5% DSS and 8 mg/kg AOM (*N* = 8 mice per group, A+D Group) and injected TRAIL for 10 times within 10 days at middle (5 week, middle T group) with 300 μg/kg dose. (**B**) After 12 weeks, colons were isolated and observed the severity of CRC. After washing the colon tissues, number of formed tumors was compared with TRAIL-treated groups. Neoplastic colon tissues were stained with H&E of each group. (**C**) HT29 colon epithelial cells were treated with different concentrations of TRAIL for 24 hr and lysed to make the protein extracts. Proteins interacting with Axin were isolated with primary antibody and detected with western blotting. (**D**) To analyze the effect of TRAIL on translocation of β-catenin, HT29 cells were treated with TRAIL for 24 hr and used for isolation of nuclear fraction. Translocated β-catenin was detected with specific antibody and Lamin B was used as normalization control.

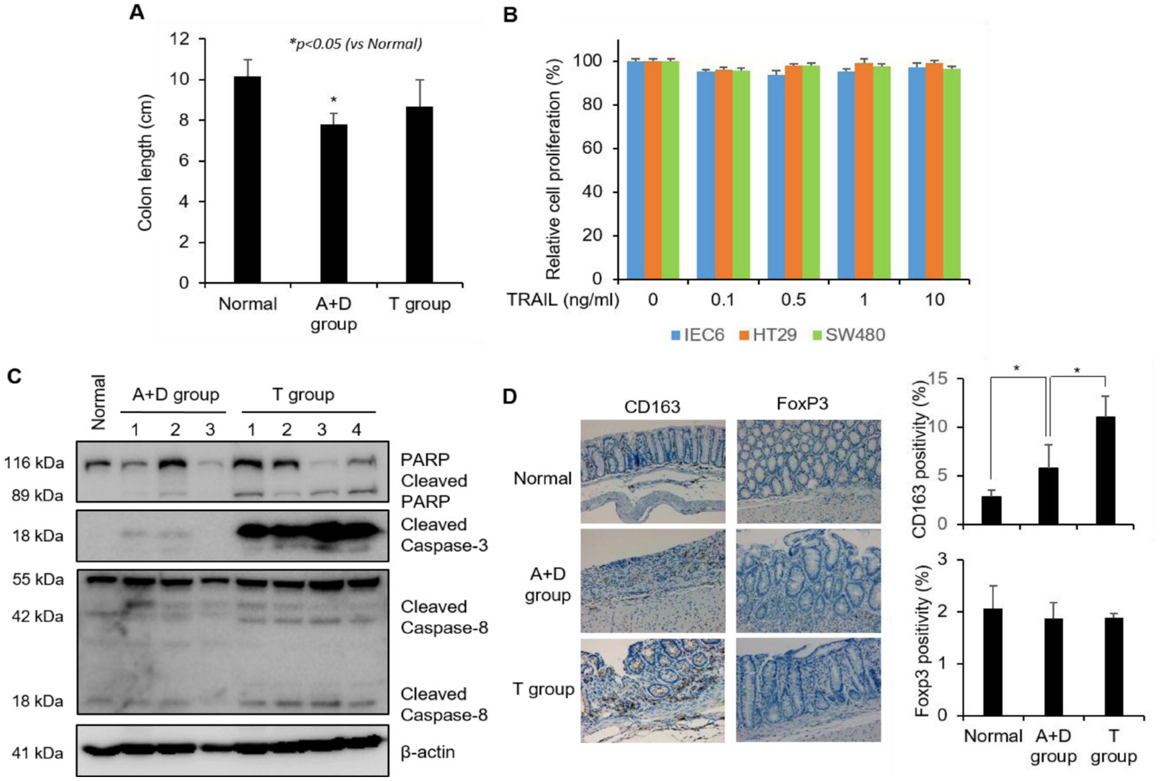

Supplementary Figure 2: TRAIL prevents pathological colitis through activation of apoptosis of intestinal epithelium. (**A**) C57BL/6 mice were treated with 2.5% DSS and 8 mg/kg AOM (*N* = 8 mice per group) and administrated TRAIL (300 μg/kg) every day. After 6 days, mice were sacrificed and colons were obtained to measure the colon length. (**B**) Colon epithelial cells (IEC6, HT29 and SW480) were seeded in 96 well plate and treated with various concentrations of TRAIL for 24 hr. Then, cell proliferation was measured with MTT assay. The relative values were presented as % of non-treated control cell. (**C**) Protein lysate were prepared from intestinal tissues after AOM/DSS treatment with/without TRAIL for 6 days. The expression level of apoptosis-related proteins were detected in animal tissues by specific PARP, Caspase-3 and Caspase-8 antibodies. The loading amount was normalized with β-actin. (**D**) To identify the subtypes of infiltrated cells, acute colitis tissues were stained with specific antibodies against CD163 (M2 macrophage) or FoxP3 (Treg). The percentage of positive staining was quantified with ImmunoRatio software and presented as mean ± SD from at least 6 fields.

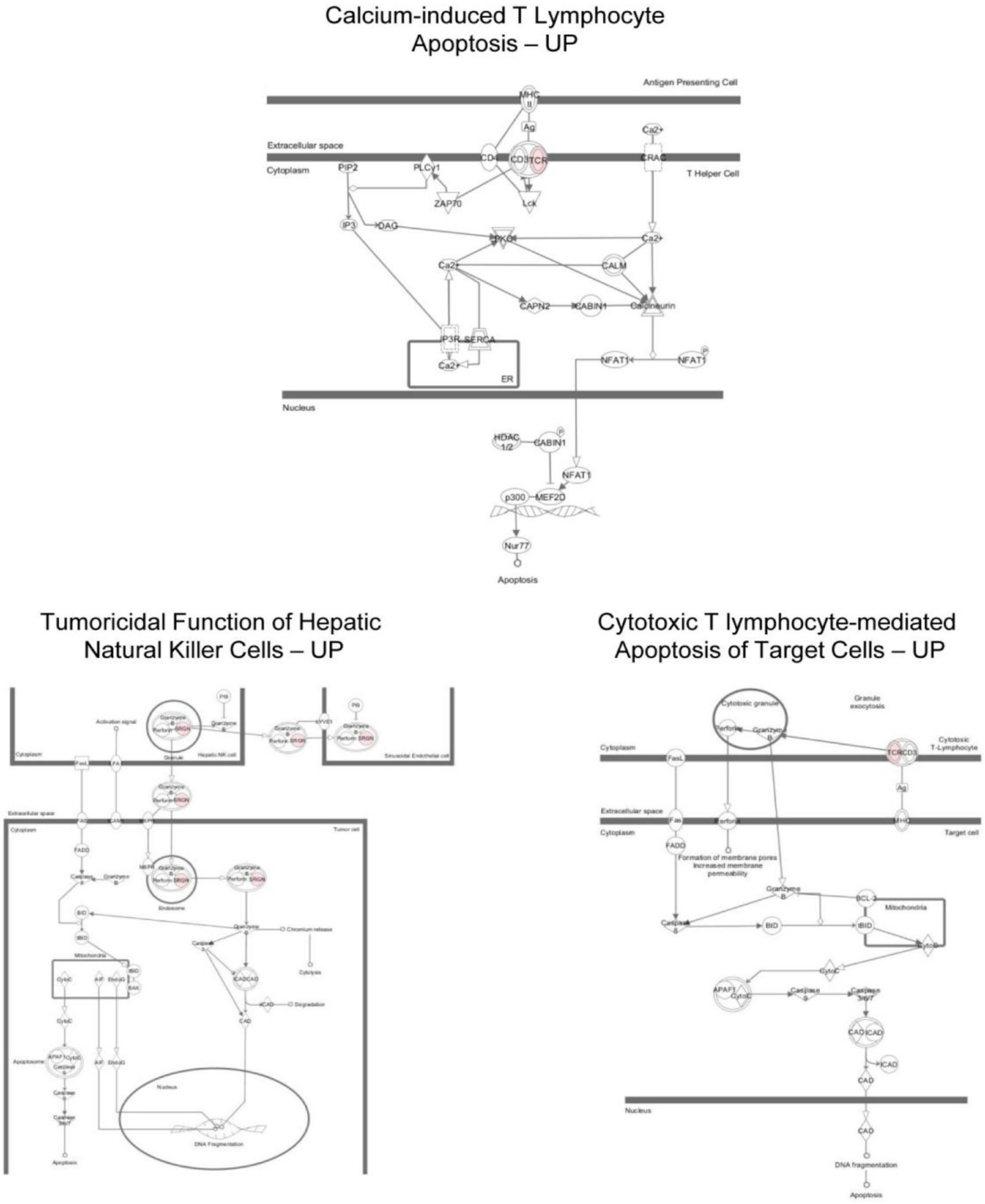

Supplementary Figure 3: TRAIL upregulates the cell death signaling. To understand the mechanism of protective effect of TRAIL in CAC, we performed the microarray using the mRNA from AOM/DSS-induced colitis model with/without TRAIL. The differentially expressed genes were analyzed with Ingenuity IPA program to identify the significantly changed pathways. Several pathways related with apoptosis of immune cells were upregulated by treatment with TRAIL.

Supplementary Table 1: List of primer sequences for qRT-PCR

qRT-PCR	Forward (5′ to 3′)	Reverse (5′ to 3′)
iNOS	CGAAACGCTTCACTTCCAA	TGAGCCTATATTGCTGTGGCT
TNF-α	CATCTTCTCAAAATTCGAGTGACAA	TGGGAGTAGACAAGGTACAACCC
IL-6	GAGGATACCACTCCCAACAGACC	AAGTGCATCATCGTTGTTCATACA
CD36	GAACCACTGCTTTCAAAAACTGG	TGCTGTTCTTTGCCACGTCA
VEGF	GGAGACTCTTCGAGGAGCACTT	GGCGATTTAGCAGCAGATATAAGAA
HGF	AAATGAGAATGGTTCTTGGTG	CTGGCCTCTTCTATGGCT
CXCL2	GGGCGGTCAAAAAGTTTGC	TGTTCAGTATCTTTTGGATGATTTTCTG
IL-10	GGTTGCCAAGCCTTATCGGA	ACCTGCTCCACTGCCTTGCT
TGF-β	CTGAACCAAGGAGACGGA	CACGTGGAGTTTGTTATC
NLRP3	GACCATCGGCCGGACTAAA	CGTCCTCGGGCTCAAACA
Caspase-1	TCCGCGGTTGAATCCTTTTCAGA	ACCACAATTGCTGTGTGTGCGCA
IL-18	CACATGCGCCTTGTGATGAC	TGCAGCCTGGGGTATTCTGT
IL-1β	GGAAGGTCCACGGGAAAGAC	AGGCAGGCAGTATCACTCATTGT
Cyclophilin	TGGAGAGCACCAAGACAGAC	TGCCGGAGTCGACAATGA
